# Post-Chimeric Antigen Receptor T-Cell Therapy Hepatitis B Virus Reactivation After 23 Months of Entecavir Prophylaxis

**DOI:** 10.14309/crj.0000000000001515

**Published:** 2024-09-12

**Authors:** Caleb J.C. McKinney, William Bigelow, Preethi G. Venkat, Neeral L. Shah

**Affiliations:** 1 Department of Internal Medicine, University of Virginia, Charlottesville, VA; 2 Division of Gastroenterology and Hepatology, University of Virginia, Charlottesville, VA

**Keywords:** hepatitis B virus, hepatitis B reactivation, oncology, immunotherapy, anti-viral prophylaxis, cellular therapy complications, CAR T-cell therapy

## Abstract

Hepatitis B virus (HBV) reactivation can occur in immunosuppressed patients. Specifically, HBV reactivation after chimeric antigen receptor T-cell (CAR T-cell) therapy is a known complication with few case reports and specific treatment guidelines. Our patient experienced HBV reactivation 27 months after CAR T-cell therapy even with 23 months of entecavir prophylaxis. This unique case highlights the need for further investigation into the risk of HBV reactivation after CAR T-cell therapy and the proper HBV prophylaxis during and after CAR T-cell therapy.

## INTRODUCTION

Hepatitis B virus (HBV) reactivation is a known complication associated with immunosuppression.^1^ In patients who develop acute HBV infection without subsequent chronic infection, reactivation may occur through immunosuppression or loss of immune control, through a rise in HBV DNA (over the patient's baseline or an absolute cutoff), or through reverse seroconversion from hepatitis B surface antigen (HBsAg) negative to positive, depending on the patient's HBsAg and total antibody to hepatitis B core antigen (anti-HBc) serology. Reactivated HBV can be categorized as HBV-associated hepatitis if alanine transaminase (ALT) increases by greater than or equal to 3 times the patient's baseline or >100 U/L.^2^

**Figure 1.**
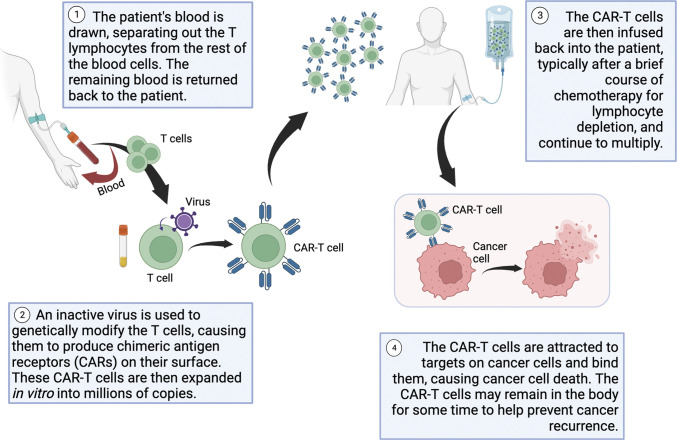
Autologous CAR T-cell therapy process.^11,12^ Created with BioRender.com. CAR T-cell, chimeric antigen receptor T-cell.

HBV reactivation and HBV-associated hepatitis have been documented in patients receiving chimeric antigen receptor T-cell (CAR T-cell) therapy, a revolutionary new pillar in cancer treatment primarily used to combat certain subsets of B-cell leukemia or lymphoma (Figure 1).^3–10^

Unfortunately, there have been few case reports describing HBV reactivation after CAR T-cell therapy, and limited safety analyses to help patients with HBV and providers understand the risks of CAR T-cell therapy. Many safety studies examining CAR T-cell therapy have excluded patients with HBV.^3–10^ Our case report demonstrates the risk of HBV reactivation after CAR T-cell therapy even with appropriate HBV prophylaxis.

## CASE REPORT

The patient is a 72-year-old man with pertinent medical history of inactive HBsAg-negative/anti-HBc-positive HBV first diagnosed in 1990 and stage IV, high-grade (3A) follicular lymphoma diagnosed in 2015.

### Background

**Figure 2.**
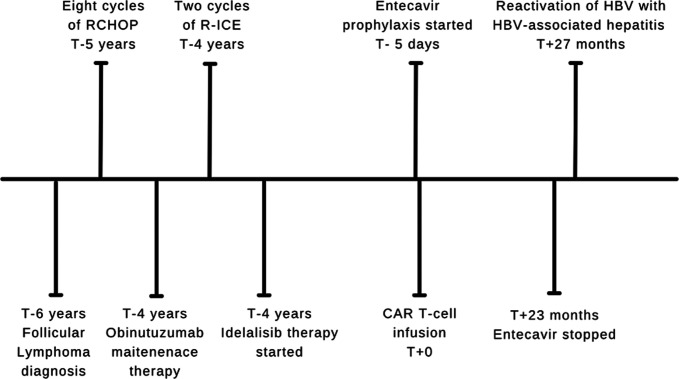
Clinical course with CAR T-cell therapy infusion as day T+0. Created with Canva. CAR T-cell, chimeric antigen receptor T-cell; HBV, Hepatitis B virus; RCHOP, rituximab, cyclophosphamide, doxorubicin hydrochloride, vincristine sulfate, and prednisone; R-ICE, rituximab, ifosfamide, carboplatin, and etoposide phosphate.

**Figure 3..**
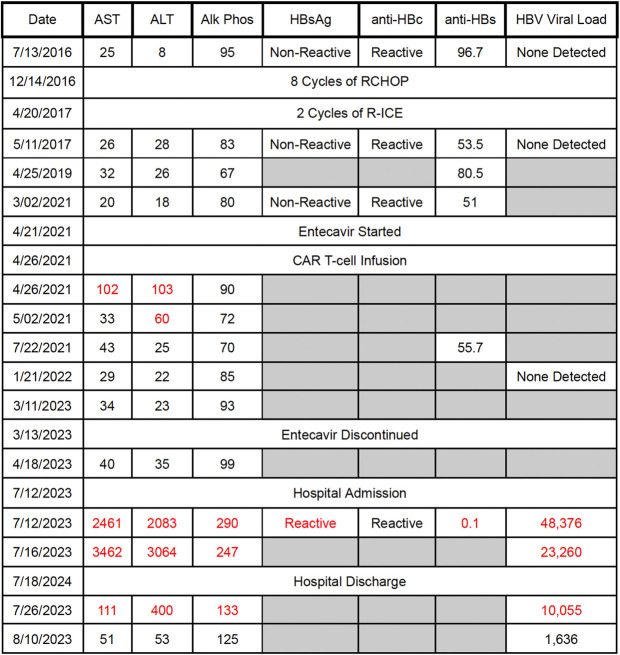
Liver function tests and hepatitis B virus laboratory test trends throughout lymphoma therapies as well as during and after hospitalization. Alk phos, alkaline phosphatase; ALT, alanine transaminase; anti-HBc, hepatitis B core antibody; anti-HBs, hepatitis B surface antibody; AST, aspartate aminotransferase; CAR T-cell, chimeric antigen receptor T-cell; HBsAg, hepatitis B surface antigen; HBV, Hepatitis B virus; RCHOP, rituximab, cyclophosphamide, doxorubicin hydrochloride, vincristine sulfate, and prednisone; R-ICE, rituximab, ifosfamide, carboplatin, and etoposide phosphate.

The patient received 8 cycles of rituximab, cyclophosphamide, doxorubicin hydrochloride, vincristine sulfate, and prednisone; 1 cycle of obinutuzumab maintenance therapy; 2 cycles of rituximab, ifosfamide, carboplatin, and etoposide phosphate; and idelalisib therapy for his follicular lymphoma (Figure 2). Before, during, and after starting these treatment regimens, the patient was HBsAg negative, anti-HBc positive, and had an undetectable HBV viral load. The patient was started on HBV prophylaxis with entecavir 0.5 mg daily 5 days before CAR T-cell therapy administration on April 26, 2021. Entecavir was discontinued in March 2023, 23 months after CAR T-cell therapy completion. A specialty pharmacy monitored the patient's compliance with entecavir via the patient's dispense history and phone calls with the patient until the medication was discontinued. As shown in Figure 3, aside from one small rise in aspartate aminotransferase (AST)/ALT directly after CAR T-cell administration, HBV and liver function test (LFT) laboratory test results remained stable during lymphoma treatments and at least 1 month after entecavir discontinuation.

### Presentation

The patient presented to an outside facility in July 2023 for lethargy and was found to have AST/ALT in the 1,000s. His symptoms persisted, and at his oncology clinic visit the following week, he was found to have a total bilirubin of 8.1 mg/dL (conjugated 6.2), AST of 2,461 U/L, ALT of 2,083 U/L, alkaline phosphatase of 290 IU/L, international normalized ratio (INR) of 1.5, and platelets of 209,000/mcL. This was 27 months after CAR T-cell therapy completion and 1 month after his computed tomography scan showing clinical remission. A liver ultrasound on the same day showed increased conspicuity of the portal triads reflecting nonspecific hepatitis.

### Hospitalization

On admission, laboratory test results showed a positive anti-HBc IgM and HBV viral load >48 k. The patient was restarted on entecavir 0.5 mg daily. The patient's hepatic function was assessed daily, and he reached a peak INR of 1.7 and total bilirubin of 10.6 mg/dL. AST peaked at 3,462 U/L, and ALT peaked at 3,348 U/L. The patient denied herbal supplement use and was taking aspirin 81 mg and atorvastatin 40 mg daily at home. He originally contracted HBV in the Navy in 1990 and denied any high-risk behavior for HBV re-exposure before admission. The patient's physical examination was notable for mild jaundice, which was improved at discharge. The patient was seen outpatient a month after discharge with improved symptoms and an HBV viral load of 1,636 IU/mL, AST 51 U/L, and ALT 53 U/L.

## DISCUSSION

Case studies have shown that with entecavir prophylaxis, patients with a history of HBV infection should generally be safe from HBV reactivation while undergoing CAR T-cell therapy. This is true for HBsAg-positive/anti-HBc-positive patients with case studies demonstrating no HBV reactivation while on prophylactic therapy.^10,13^ One case series did show reactivation, but only in 1 of 19 HBsAg-positive/anti-HBc-positive patients (5.3%) on entecavir prophylaxis.^3^ Case reports have also shown that patients with HBsAg-negative/anti-HBc-positive serologies have an incredibly low risk of HBV reactivation, even without HBV prophylaxis. In one study, very few HBsAg-negative/anti-HBc-positive patients (5.4%) undergoing CAR T-cell therapy were started on any infectious prophylaxis, and all patients avoided hepatitis B reactivation.^3^

Current guidelines from the American Association for the Study of Liver Diseases (AASLD)state that HBsAg-negative, anti-HBc-positive patients should be started on prophylaxis for HBV when given treatments that target B lymphocytes, such as CAR T-cell therapy.^2^ HBV prophylaxis should be started either as soon as possible before, or in conjunction with, the onset of immunosuppressive therapy and should be continued for 12 months afterward for patients undergoing anti-CD20 therapies. Entecavir has consistently been a preferred drug for HBV prophylaxis.^2^ At this time, no specific HBV prophylaxis guidelines have been tailored to address the myriad unique CAR T-cell therapy regimens such as the JCAR017 CAR T-cell therapy (B-lymphocyte, CD19-directed therapy) our patient received.

Interestingly, our HBsAg-negative/anti-HBc-positive patient was started on entecavir prophylaxis before starting CAR T-cell therapy and prophylaxis was continued for 23 months after therapy. Despite his low-risk serology and properly administered entecavir prophylaxis, our patient developed HBV reactivation and HBV-associated hepatitis.

Preimmunosuppression anti-HBs titers have been suggested to be helpful in predicting risk of HBV reactivation.^14–18^ Some studies have shown the potential for reducing the risk of HBV reactivation by administering an HBV vaccine before anti-B-cell therapies.^19^ The potential benefit in providing patients who are undergoing CAR T-cell therapy with an HBV vaccine remains unknown, but could be a promising option requiring further investigation, specifically for patients with pretherapy anti-HBs <100 mIU/mL.

Our patient far exceeded the AASLD guidelines for HBV prophylaxis surrounding CAR T-cell therapy, but still developed HBV reactivation and HBV-associated hepatitis. This may be due to the presence of inactive CAR T cells that are prone to robust reactivation and transaminase elevation despite prophylaxis. An alternative explanation could be a very late HBV reactivation from a previous rituximab-containing therapy regimen where the patient did not receive HBV prophylaxis; however, the lengthy time course and normal HBV serologies and LFTs intervally before CAR T-cell therapy make this explanation less likely.

Our recommendation for patients with a history of HBV undergoing CAR T-cell therapy would be to measure LFTs every 3 to 6 months after discontinuation of prophylaxis, with prompt evaluation of HBV serologies following any changes in LFTs. This could expedite treatment for patients and potentially avoid complications of HBV reactivation. Further investigation is needed to understand the true risk for HBsAg-negative, anti-HBc-positive patients undergoing CAR T-cell therapy even with appropriate HBV prophylaxis.

## DISCLOSURES

Author contributions: CJC McKinney: data acquisition; writing (original draft). W. Bigelow: writing (review & editing); final approval. PG Venkat: writing (review & editing); Final approval. NL Shah: conceptualization, supervision, writing (review & editing), Final approval. N.L. Shah is the article guarantor.

Financial disclosure: None to report.

Informed consent was obtained for this case report.

## References

[1] Palmore TN Shah NL Loomba R Reactivation of hepatitis B with reappearance of hepatitis B surface antigen after chemotherapy and immunosuppression. Clin Gastroenterol Hepatol. 2009;7(10):1130–7. 19577007 10.1016/j.cgh.2009.06.027 PMC2779698

[2] Terrault NA Lok ASF McMahon BJ Update on prevention, diagnosis, and treatment of chronic hepatitis B: AASLD 2018 hepatitis B guidance. Hepatology. 2018;67(4):1560–99. 29405329 10.1002/hep.29800 PMC5975958

[3] Cao W Wei J Wang N Entecavir prophylaxis for hepatitis B virus reactivation in patients with CAR T-cell therapy. Blood. 2020;136(4):516–9. 32291456 10.1182/blood.2020004907

[4] Han L Zhou J Zhou K Safety and efficacy of CAR-T cell targeting BCMA in patients with multiple myeloma coinfected with chronic hepatitis B virus. J Immunother Cancer. 2020;8(2):e000927. 32792360 10.1136/jitc-2020-000927 PMC7430488

[5] Ma Y Yang L Bao Y Yang Y Chen L Zheng M. Case Report: Post-CAR-T infusion HBV reactivation in two lymphoma patients despite entecavir preventive therapy. Front Immunol. 2021;12:751754. 34691067 10.3389/fimmu.2021.751754 PMC8535441

[6] Wei J Zhu X Mao X Huang L Meng F Zhou J. Severe early hepatitis B reactivation in a patient receiving anti-CD19 and anti-CD22 CAR T cells for the treatment of diffuse large B-cell lymphoma. J Immunother Cancer. 2019;7(1):315. 31753002 10.1186/s40425-019-0790-y PMC6868854

[7] Mustafayev K Torres H. Hepatitis B virus and hepatitis C virus reactivation in cancer patients receiving novel anticancer therapies. Clin Microbiol Infect. 2022;28(10):1321–7. 35283317 10.1016/j.cmi.2022.02.042

[8] Wang Y Liu Y Tan X Safety and efficacy of chimeric antigen receptor (CAR)-T-cell therapy in persons with advanced B-cell cancers and hepatitis B virus-infection. Leukemia. 2020;34(10):2704–7. 32594100 10.1038/s41375-020-0936-4

[9] Yang C Xie M Zhang K Risk of HBV reactivation post CD19-CAR-T cell therapy in DLBCL patients with concomitant chronic HBV infection. Leukemia. 2020;34(11):3055–9. 32533094 10.1038/s41375-020-0913-y

[10] Strati P Nastoupil LJ Fayad LE Samaniego F Adkins S Neelapu SS. Safety of CAR T-cell therapy in patients with B-cell lymphoma and chronic hepatitis B or C virus infection. Blood. 2019;133(26):2800–2. 31101626 10.1182/blood.2019000888 PMC7265784

[11] Chimeric antigen receptor (CAR) T-cell therapy (https://www.lls.org/treatment/types-treatment/immunotherapy/chimeric-antigen-receptor-car-t-cell-therapy). Accessed August 31, 2023.

[12] Martínez Bedoya D Dutoit V Migliorini D. Allogeneic CAR T Cells: An alternative to overcome challenges of CAR T cell therapy in glioblastoma. Front Immunol 2021;12:640082. 33746981 10.3389/fimmu.2021.640082 PMC7966522

[13] Liu W Huang W Wang M Risk of hepatitis B reactivation is controllable in patients with B-cell lymphoma receiving anti-CD19 CAR T cell therapy. Br J Haematol. 2020;191(1):126–9. 32671820 10.1111/bjh.16951

[14] Cho Y Yu SJ Cho EJ High titers of anti-HBs prevent rituximab-related viral reactivation in resolved hepatitis B patient with non-Hodgkin's lymphoma. J Med Virol. 2016;88(6):1010–7. 26531242 10.1002/jmv.24423

[15] Matsue K Kimura S Takanashi Y Reactivation of hepatitis B virus after rituximab-containing treatment in patients with CD20-positive B-cell lymphoma. Cancer. 2010;116(20):4769–76. 20597091 10.1002/cncr.25253

[16] Pei SN Ma MC Wang MC Analysis of hepatitis B surface antibody titers in B cell lymphoma patients after rituximab therapy. Ann Hematol. 2012;91(7):1007–12. 22273839 10.1007/s00277-012-1405-6

[17] Seto WK Chan TS Hwang YY Hepatitis B reactivation in patients with previous hepatitis B virus exposure undergoing rituximab-containing chemotherapy for lymphoma: A prospective study. J Clin Oncol. 2014;32(33):3736–43. 25287829 10.1200/JCO.2014.56.7081

[18] Yeo W Chan TC Leung NW Hepatitis B virus reactivation in lymphoma patients with prior resolved hepatitis B undergoing anticancer therapy with or without rituximab. J Clin Oncol. 2009;27(4):605–11. 19075267 10.1200/JCO.2008.18.0182

[19] Araujo-Neto JM Guimarães GS Fernandes FF Soares MA. Hepatitis B surface antibody (anti-HBs) kinetics during rituximab chemotherapy and performance of hepatitis B vaccine before immunosuppression: Two prospective studies. Viruses. 2022;14(8):1780. 36016402 10.3390/v14081780 PMC9415137

